# Three-year nationwide survey of microbiology laboratory equipment conditions in China’s CDC: setting new benchmarks

**DOI:** 10.3389/fpubh.2025.1679072

**Published:** 2026-01-13

**Authors:** Peihua Niu, Zhenlu Sun, Yu Zhang, Dapeng Zhang, Ruiqing Zhang, Yiming Zhao, Fengyu Tian, Jieqiong Zhang, Hongmei Zheng, Jianqiang Guo, Ping Cheng, Xuejun Ma, Ji Wang

**Affiliations:** 1 National Key Laboratory of Intelligent Tracking and Forecasting for Infectious Diseases, NHC Key Laboratory of Medical Virology and Viral Diseases, National Institute for Viral Disease Control and Prevention, Chinese Center for Disease Control and Prevention, Beijing, China; 2 Yantai Center for Disease Control and Prevention, Yantai, China

**Keywords:** CDC, China, laboratory equipment, microbiology laboratories, penetration rate, public health

## Abstract

**Background:**

This study evaluates the penetration and evolution of laboratory equipment in China’s Centers for Disease Control and Prevention (CDCs) from 2021 to 2023, establishing benchmarks for optimal equipment configuration and operational capacity enhancement.

**Methods:**

Data were collected from CDCs at provincial, municipal, and county levels over three years using stratified sampling and structured questionnaires. These captured information on equipment penetration and user satisfaction. Statistical analyses, including Chi-square tests, ANOVA, and Fisher’s exact tests, identified trends and disparities in equipment distribution across administrative tiers.

**Results:**

Analysis of 540 questionnaires from 376 CDCs showed that essential equipment like Biological Safety Cabinets (BSCs) and qPCR machines achieved over 95% penetration across all levels. Municipal CDCs reported the largest increases in equipment acquisition, followed by provincial CDCs, with minimal changes at the county level. The penetration of domestic qPCR machines rose from 21.8% in 2021% to 28.3% in 2023, with domestic brands closing the gap with international counterparts. Post-COVID-19, strategies for optimizing equipment utilization appear promising.

**Conclusion:**

This study provides a valuable dataset on microbiology laboratory equipment in China’s CDCs, revealing distinct patterns in equipment retention and acquisition. Disparities in qPCR machine and High-throughput Sequencer (HTS) penetration and satisfaction rates highlight a shift toward domestic manufacturing. The findings offer essential benchmarks for optimizing laboratory infrastructure and enhancing public health responses.

## 1 Background

Microbiology laboratories (Micro-Labs) serve a pivotal role in the prevention, control, and response to infectious diseases by significantly enhancing the capacity for detecting and identifying pathogens pertinent to both public and global health security (1). The condition and functionality of laboratory equipment constitute a core aspect of these facilities (2). This is particularly evident during pandemics such as COVID-19, where state-of-the-art laboratory instruments are indispensable for the etiological diagnosis of pathogens, patient monitoring, and epidemiological surveillance, thereby ensuring the timely and precise detection and management of infectious diseases (3).

The Centers for Disease Control and Prevention (CDC) at the provincial, municipal, and county levels in China serve as the principal agencies in managing the challenges posed by emerging and re-emerging infectious diseases (4). A thorough evaluation of the hardware infrastructure in the Micro-Labs of these CDCs is imperative to ascertain their compliance with established standards. Moreover, correlating these hardware assessments with data pertaining to laboratory personnel and their capabilities is critical for identifying key areas where the overall operational capacity of these laboratories can be enhanced (5).

Micro-Labs are equipped with a comprehensive array of instruments that are indispensable for the prevention and control of infectious diseases. These instruments, encompassing categories such as biosafety, centrifugation, nucleic acid amplification, immunological assays, sequencing, and automation, exhibit distribution patterns and penetration rates that are closely aligned with the operational mandates of CDCs across different administrative levels (5, 6). The COVID-19 pandemic has brought into sharp relief the significance of certain essential equipment, particularly Biological Safety Cabinets (BSC) and quantitative real-time PCR machines (qPCR machine), which are integral to optimizing the nucleic acid testing capabilities of the CDCs. High-throughput Sequencers (HTS) have emerged as pivotal tools in the detection and comprehensive analysis of microbial genomes (7), Consequently, it is imperative to conduct a rigorous quantification of these instruments and perform an in-depth evaluation of their operational performance and perceived reputation within laboratory settings.

In the aftermath of the SARS outbreak, national cross-sectional surveys conducted in 2006 and 2013 indicated a substantial increase in the allocation rate of Class A equipment within China’s CDC system, rising from 28.1% to 65.0% (8). Before the onset of the COVID-19 pandemic, research had already undertaken a comprehensive questionnaire survey assessing equipment management and sustainability across 227 veterinary diagnostic laboratories spanning 136 countries (9). During the COVID-19 pandemic, our study examined the penetration rates of Micro-Labs equipment within CDCs at the provincial, municipal, and county levels in 2021 (5), and we have systematically continued this survey over the ensuing two years. To our knowledge, there has not been a precedent for a nationwide survey of this scope and duration, sustained over a consecutive three-year period.

The aims of this study are multifaceted: 1. To systematically investigate the penetration rates of Micro-Labs equipment across the various tiers of CDCs in China from 2021 to 2023 and to rigorously analyze the trends in these rates over time. 2. To quantify the precise numbers of Three-zone PCR Laboratories (PCR Lab), BSCs, and qPCR machines, thereby deriving optimal configuration standards from this data. 3. To assess the penetration rates and user satisfaction of different brands and models of qPCR machines and HTS, and to analyze the shifts in their domestic market share over this three-year span. 4. To explore preliminary strategies for the management of surplus equipment in the post-COVID-19 landscape.

## 2 Methods

### 2.1 Study participants and data collection

A stratified sampling design was applied following the standardized framework used in previous nationwide CDC surveys on biosafety and laboratory capacity (5, 10). The survey was conducted annually from 2021 to 2023, with each provincial CDC coordinating participation across provincial, municipal, and county levels. Each year, approximately ten qualified respondents were nominated per province, ensuring representation from different administrative levels and institutional types. Selection criteria emphasized diversity in professional title, role, and academic qualification, thereby achieving balanced representativeness across the 31 provincial administrative divisions. Data were collected through a structured questionnaire comprising six multiple-choice questions, three single-choice question, and one open-ended question. These data were compiled into a comprehensive dataset referred to as the “respondents database.” From this dataset, a non-redundant subset was meticulously extracted to form the “institute database,” which served as the foundation for this study and reflects the institutional characteristics of the CDCs rather than individual responses.

### 2.2 Data screening and validation

To ensure that each CDC was represented by a single, high-quality questionnaire annually, records from the same unit were meticulously screened, retaining only the most exemplary submission. Selection criteria favored responses from individuals with senior professional titles, extensive service duration, and optimal response times. When necessary, direct consultation with the heads of provincial CDC Micro-Labs was conducted to ascertain the most reliable data source. The data screening workflow is illustrated in Supplementary Figure S1.

For the multiple-choice questions concerning the models of qPCR machines and HTS, records were excluded from the institute database if the models marked as satisfactory were not also marked as owned. Among all surveyed models of qPCR machines and HTS, those with no reported satisfaction or with a penetration rate below ten units were omitted from consideration. Only the models that met these criteria were eligible for inclusion in the satisfaction evaluation.

### 2.3 Statistical methods

This study evaluates laboratory equipment conditions by examining the penetration rate of various Micro-Labs instruments, defined as (number of CDCs with the equipment/total number of surveyed CDCs) × 100%. Chi-square tests or Fisher’s exact tests were applied to assess categorical variables, such as disparities in equipment ownership across CDCs of varying administrative levels. For continuous variables, such as differences in the number of BSCs among CDC levels, one-way analysis of variance (ANOVA) was performed. Data normality and variance homogeneity were tested using the Shapiro–Wilk and Levene’s tests; when assumptions were violated, Welch ANOVA or Kruskal-Wallis tests were applied. Multiple comparisons were adjusted using the Bonferroni correction. Because institutional identifiers were anonymized, each year’s dataset was analyzed as an independent cross-sectional sample. A *p*-value of <0.05 was considered statistically significant.

The recommended standards for the number of PCR Lab, BSCs, and qPCR machines within CDCs at various levels are articulated as “mean (reasonable range).” This reasonable range is determined through a synthesis of the interquartile range and standard deviation methods, specifically selecting the two values within these parameters that are closest to the mean.

### 2.4 User satisfaction metrics

User Satisfaction (US) and Weighted Satisfaction (WS) metrics were employed to assess the level of satisfaction among CDC users with specific models of qPCR and HTS. US is defined as the proportion of satisfied users relative to the total number of users, calculated by the formula: US = (number of satisfied users / total number of users) × 100%. The 95% confidence interval for US is determined using the Clopper-Pearson method, which provides an exact binomial confidence interval. WS further refines this measure by incorporating the penetration rate as a weighting factor, calculated as WS = (US × penetration rate) × 100%.

### 2.5 Subjective response analysis

Responses to the subjective questions were meticulously compiled, with common words filtered out to calculate word frequencies. The analysis of word frequency results facilitated the categorization of each response into distinct thematic groups, which were then ranked by their frequency of occurrence.

### 2.6 Naming convention

To avoid potential disputes, this study uses a naming convention that conceals the true identities of qPCR and HTS brands and models with 2–3 character codes. The first character—'Q’ for qPCR machines and ‘S’ for HTS —indicates the device type, with lowercase letters signifying domestic equipment. The second character is a letter denoting the brand, while QA and SA do not represent the same brand. The third character is a numeral indicating the model, which is omitted If a brand predominantly markets a single model. This randomized naming system ensures no association or implication with actual brands or models.

## 3 Results

### 3.1 Demographics and data distribution

The analysis conducted in this study was based on an institute database consisting of 540 meticulously selected questionnaires, derived from an initial respondents database of 990 questionnaires. A total of 376 CDCs engaged in this three-year longitudinal survey, with 31 CDCs (8.24%) maintaining participation across all three years, 102 CDCs (27.13%) participating for two years, and 243 CDCs (64.63%) participating for one year. Importantly, no questionnaires were sourced from the same CDC within the same year, ensuring the independence of each year’s data set.

Out of the 540 questionnaires analyzed, 239 (44.26%) were collected in 2021, 177 (32.78%) in 2022, and 124 (22.96%) in 2023. The distribution among the levels of CDCs was as follows: 74 (13.70%) from provincial CDCs, 396 (73.33%) from municipal CDCs, and 70 (12.96%) from county CDCs. The provinces with the highest participation rates were Jiangxi with 69 responses (12.78%), Hunan with 29 (5.37%), Jiangsu with 27 (5.00%), Guizhou with 27 (5.00%), and Heilongjiang with 22 (4.07%).

Statistical assumptions were verified before comparative analyses. Data followed normal and homogeneous distributions in most variables; where assumptions were violated, Welch ANOVA or Kruskal-Wallis tests produced consistent results. All reported *p*-values were Bonferroni-adjusted where multiple comparisons were performed.

### 3.2 Equipment penetration rate

**Figure 1:**
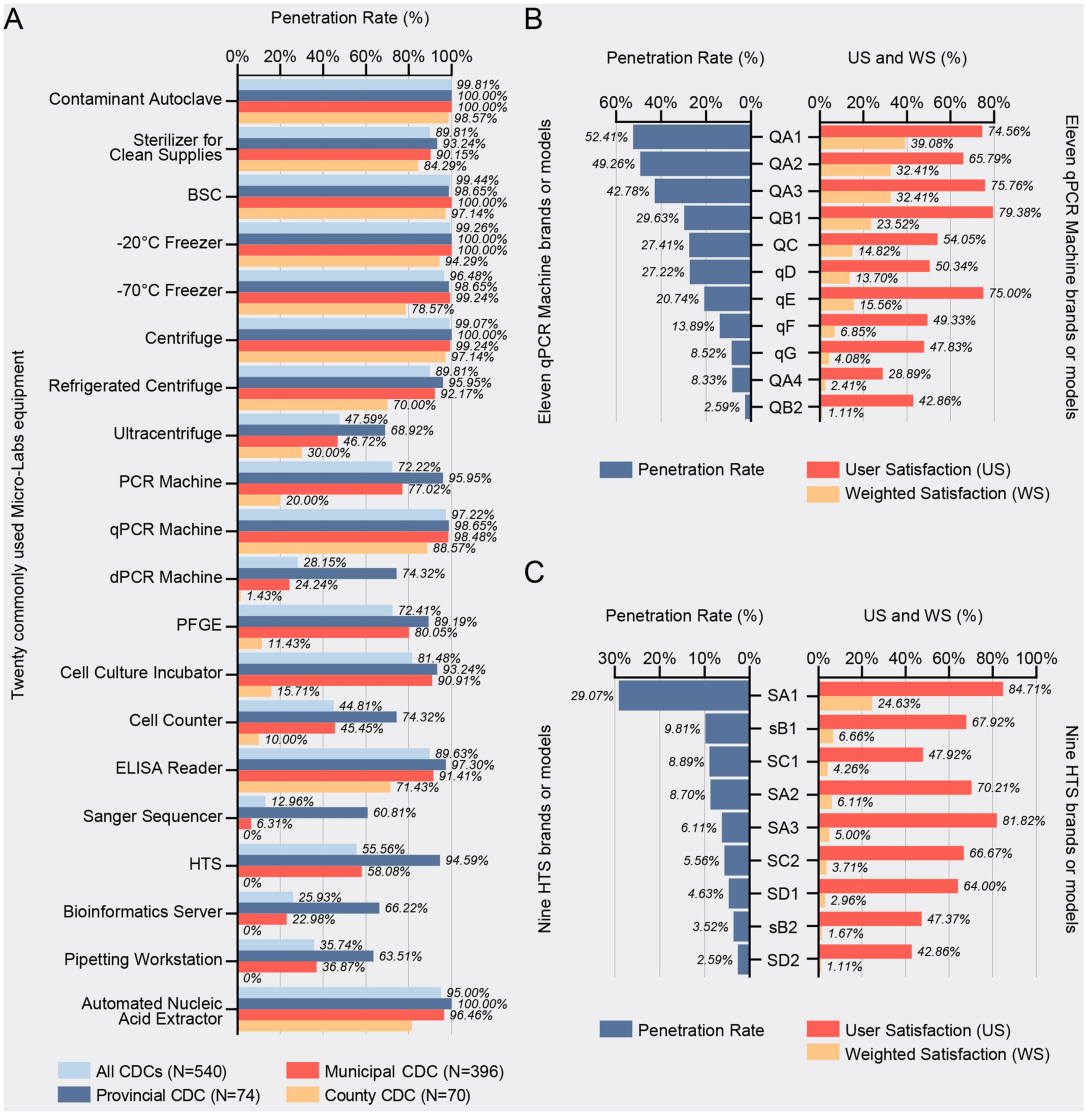
Penetration and satisfaction rates of laboratory equipment across China’s CDCs. **(A)** Aggregated penetration rates of 20 commonly used microbiology laboratory equipment items across provincial, municipal, and county CDCs, compiled from data spanning 2021–2023 (*N* = 540 CDCs; Provincial = 74, Municipal = 396, County = 70). Absolute numbers of CDCs owning each equipment item are listed in Supplementary Table 1. **(B)** Penetration rate, user satisfaction (US), and weighted satisfaction (WS) for 11 different qPCR machine brands or models, with lowercase “q” indicating domestic brands. **(C)** Penetration rate, US and WS for nine high-throughput sequencer (HTS) brands or models, with lowercase “s” indicating domestic brands.

A total of 20 commonly used Micro-Labs equipment types were surveyed over the three-year period (Figure 1A). The penetration rates, ranked from highest to lowest, were as follows: Contaminant Autoclave at 99.81% (539/540), BSC at 99.44% (537/540), −20 °C Freezer at 99.26% (536/540), Centrifuge at 99.07% (535/540), qPCR Machine at 97.22% (525/540), −70 °C Freezer at 96.48% (521/540), Automated Nucleic Acid Extractor at 95.00% (513/540), Sterilizer for Clean Supplies at 89.81% (485/540), Refrigerated Centrifuge at 89.81% (485/540), ELISA Reader at 89.63% (484/540), Cell Culture Incubator at 81.48% (440/540), Pulsed-Field Gel Electrophoresis (PFGE) at 72.41% (391/540), PCR Machine at 72.22% (390/540), HTS at 55.56% (300/540), Ultracentrifuge at 47.59% (257/540), Cell Counter at 44.81% (242/540), Pipetting Workstation at 35.74% (193/540), Digital PCR Machine (dPCR Machine) at 28.15% (152/540), Bioinformatics Server at 25.93% (140/540), and Sanger Sequencer at 12.96% (70/540).

When comparing provincial and municipal CDCs, significant disparities were observed in the penetration rates of several key pieces of equipment, including Sanger Sequencers, dPCR machines, PCR machines, Bioinformatics Servers, Pipetting Workstations, Cell Counters, Ultracentrifuges, and HTS. In addition, significant differences in equipment penetration rates were identified between municipal and county CDCs across all categories, except for Centrifuges, Contaminant Autoclaves, and Sterilizers for Clean Supplies (Figure 1A).

### 3.3 Annual trends in equipment acquisition

An analysis of equipment acquisition reveals notable trends in the period between 2021 and 2022. Provincial CDCs exhibited substantial enhancements with the acquisition of Bioinformatics Servers, increasing from 50.00% to 84.62%, Pipetting Workstations, which rose from 46.43% to 76.92%, and Ultracentrifuges, expanding from 53.57% to 84.62%. Municipal CDCs also demonstrated significant growth in their equipment base, with Bioinformatics Servers rising from 15.00% to 24.63%, Ultracentrifuges from 39.38% to 51.49%, and HTS from 39.38% to 61.19%. In contrast, County CDCs did not record any significant additions to their equipment inventory.

In the subsequent period from 2022 to 2023, Provincial CDCs showed no notable changes in equipment acquisition. However, Municipal CDCs further augmented their capabilities with the addition of dPCR machines, increasing from 23.13% to 35.29%, Cell Counters from 38.81% to 58.82%, Refrigerated Centrifuges from 87.31% to 95.10%, and HTS from 61.19% to 83.33%. County CDCs, once again, did not report any significant new acquisitions during this period.

### 3.4 Disparities and recommendations for PCR labs, BSCs, and qPCR machines

Significant disparities were observed in the number of PCR Labs, BSCs, and qPCR machines across different levels of CDCs. Provincial CDCs exhibited higher averages with 3.62 PCR labs, 7.68 BSCs, and 11.89 qPCR machines compared to municipal (2.26, 4.74, and 8.29, respectively) and county levels (1.63, 2.00, and 2.51, respectively). Median values mirrored these trends, showing 3, 6, and 10 for provincial, 2, 4, and 6 for municipal, and 1, 2, and 2 for county levels, respectively. Notably, while the distribution of PCR labs and BSCs remained consistent over the years, significant temporal variation was observed in the distribution of qPCR machines, with median values increasing from 4 in the initial year to 7 in subsequent years, highlighting a dynamic shift in resource allocation.

Descriptive statistical analysis over the three-year period provides the following recommendations for equipment allocation: Provincial CDCs should maintain an average of 3.62 PCR Labs (2.08–5.16), 7.68 BSCs (4.47–10.24), and 11.89 qPCR Machines (7.68–13.24). For municipal CDCs, the recommended allocations are 2.26 PCR Labs (1.03–3.50), 4.74 BSCs (2.26–8.29), and 8.29 qPCR Machines (4.74–9.09). Meanwhile, county CDCs should be equipped with 1.63 PCR Labs (0.73–3.25), 2.00 BSCs (1.63–2.00), and 2.51 qPCR Machines (2.00–2.51). These guidelines reflect the optimal distribution of resources to support effective laboratory operations at each administrative level.

**Figure 2:**
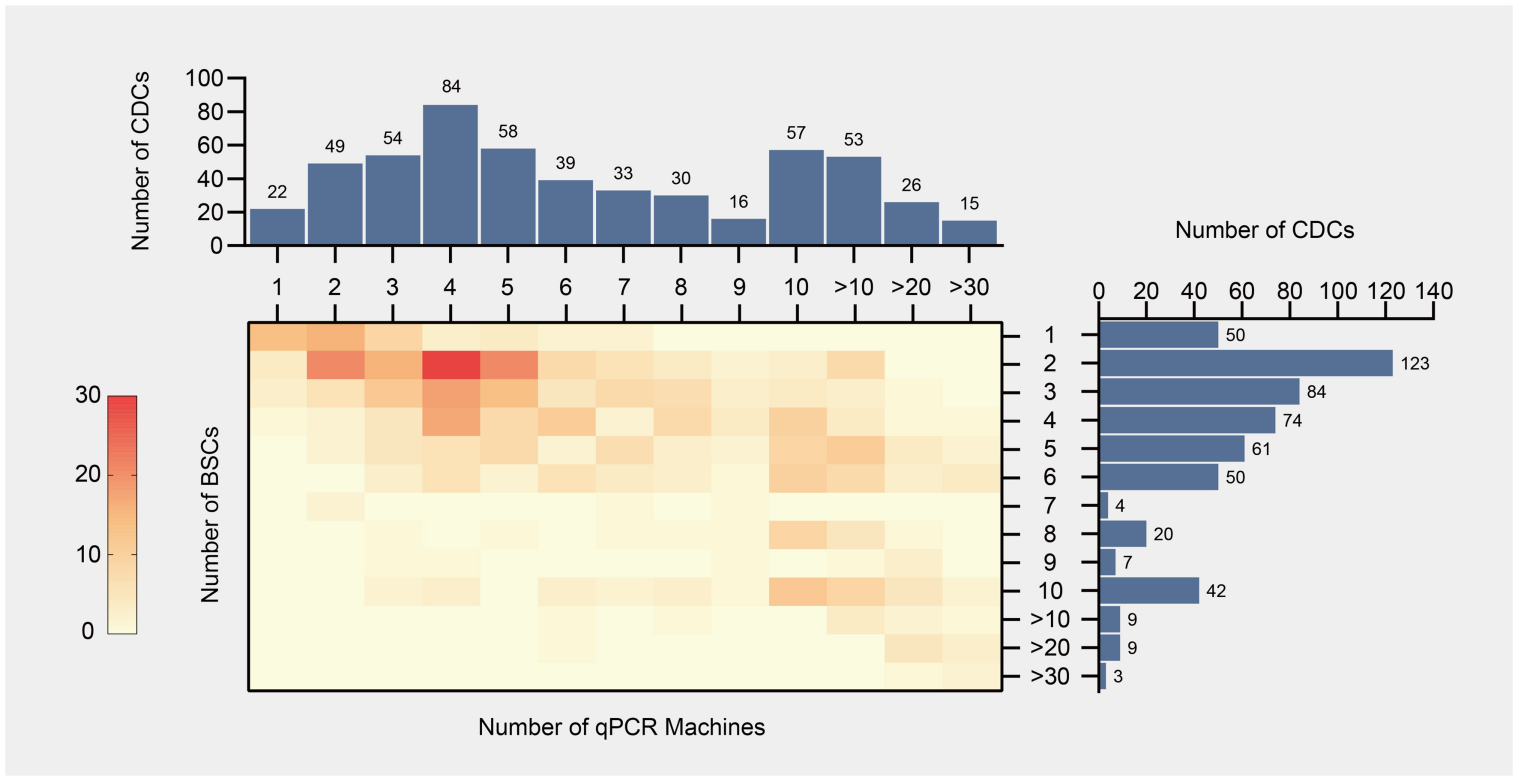
Distribution of qPCR machines and biological safety cabinets (BSCs) across China’s CDCs. The heatmap shows the joint distribution of qPCR machines and BSCs in CDCs nationwide, compiled from data collected between 2021 and 2023 (*N* = 536). Color intensity indicates the number of CDCs in each category, with a standardized scale displayed on the left. The accompanying bar charts present the numbers of CDCs with specific quantities of qPCR machines (top) and BSCs (right).

The configuration most frequently observed consisted of two BSCs in 123 CDCs and four qPCR machines in 84 CDCs, with a combined setup of two BSCs and four qPCR machines identified in 30 CDCs (Figure 2). A Pearson correlation coefficient of 0.62 was calculated, indicating a positive correlation between the quantities of BSCs and qPCR machines across the surveyed CDCs.

### 3.5 Penetration rate and satisfaction of qPCR machines

In evaluating 27 distinct models of qPCR machines, 7 models were universally rated as unsatisfactory. Of the remaining 20 models, 9 had a penetration rate of fewer than 10 units, excluding them from further analysis. Consequently, only 11 models met the criteria for inclusion in penetration rate and user satisfaction assessments (Figure 1B).

The penetration rates for the 11 models of qPCR machines, ranked from highest to lowest, are as follows: QA1 at 52.41% (283/540), QA2 at 49.26% (266/540), QA3 at 42.78% (231/540), QB1 at 29.63% (160/540), QC at 27.41% (148/540), qD at 27.22% (147/540), qE at 20.74% (112/540), qF at 13.89% (75/540), qG at 8.52% (46/540), QA4 at 8.33% (45/540), and QB2 at 2.59% (14/540). Notably, the top five models for both US and WS are identical, comprising the QB1, QA3, qE, QA1, and QA2. Among these, qE stands out as a domestic brand.

### 3.6 Penetration rate and satisfaction of HTS

Among the 11 models of HTS evaluated, one model was uniformly deemed unsatisfactory. Of the remaining 10 models, one exhibited a penetration rate below 10 units, rendering it statistically insignificant for further analysis. Consequently, only 9 models were deemed eligible for calculating penetration rates and underwent satisfaction evaluation (Figure 1C).

Among the 9 HTS models assessed, penetration rates varied, with the SA1 leading at 29.07% (157/540), followed by the sB1 at 9.81% (53/540), and the SC1 at 8.89% (48/540). The SA2 registered at 8.70% (47/540), while the SA3 achieved 6.11% (33/540). Additional models included the SC2 at 5.56% (30/540), SD1 at 4.63% (25/540), sB2 at 3.52% (19/540), and SD2 at 2.59% (14/540). Notably, four models ranked among the top in both US and WS indices: SA1, SA3, SA2, and sB1, with sB1 distinguishing itself as a domestic brand.

### 3.7 Domestic adoption of qPCR machines and HTS

**Figure 3:**
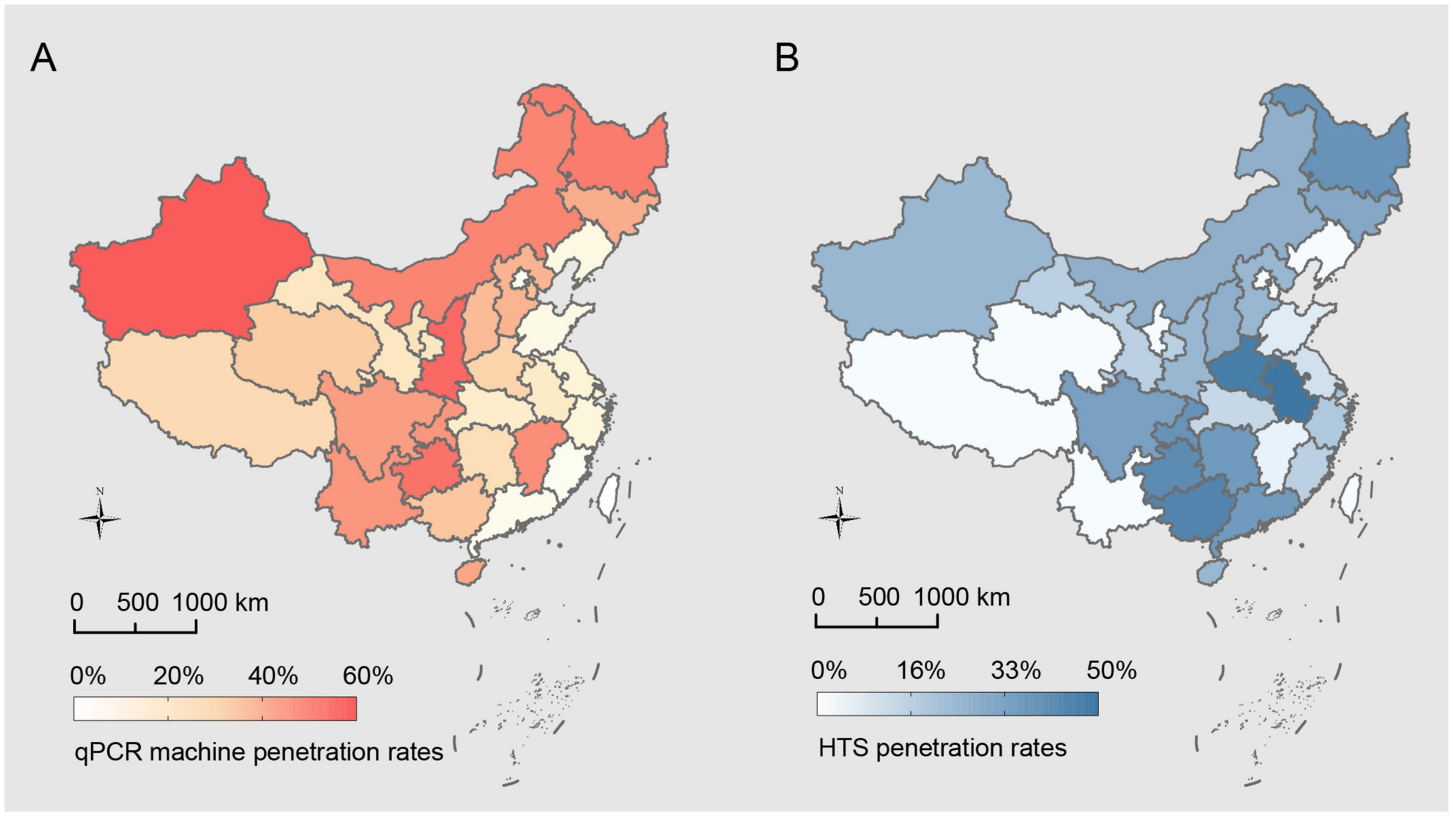
Geographic distribution of domestic equipment penetration in China’s CDCs. **(A)** Geographic distribution of domestic qPCR machine penetration rates across all administrative levels (provincial, municipal, and county), compiled from data spanning 2021–2023. Penetration rates range from 5.56% to 60.71%, with color intensity representing the proportion of CDCs equipped with domestic qPCR machines. **(B)** Geographic distribution of domestic high-throughput sequencer (HTS) penetration rates, ranging from 0% to 50.00%, with color intensity representing the proportion of CDCs equipped with domestic HTS.

Out of the 27 qPCR machine models surveyed, 11 models are domestically manufactured, accounting for 40.74% (11/27) of the total models evaluated. The domestic penetration rate stands at 25.37% (399/1573), varying significantly across provinces, ranging from 5.56 to 60.71% (Figure 3A). The data indicates a year-on-year increase in the penetration rates of domestic qPCR machines: 21.84% (145/664) in 2021, 27.72% (148/534) in 2022, and 28.27% (106/375) in 2023.

Of the 11 HTS models evaluated, 2 are domestically manufactured, constituting 18.18% (2/11) of the models assessed, with an overall domestic penetration rate of 16.67% (72/432), which varies significantly across provinces, ranging from 0 to 50.00% (Figure 3B). The penetration rate for domestic HTS models increased from 15.22% (21/138) in 2021 to 17.47% (29/166) in 2022. In 2023, the rate was 17.19% (22/128), maintaining a level nearly identical to the previous year.

### 3.8 Management of idle testing equipment post-COVID-19

The 2023 survey uniquely addressed inquiries concerning the presence and management of idle testing equipment in the aftermath of COVID-19. Out of 124 CDCs surveyed, 53 (42.74%) reported the presence of idle equipment, while 55 (44.35%) indicated no idle equipment. An additional 16 (12.90%) were uncertain regarding the status of their equipment. Statistical analysis revealed no significant differences among provincial, municipal, and county CDCs in terms of reported equipment idleness.

A total of 43 questionnaires offered recommendations for the management of idle testing equipment, which were analyzed for word frequency and thematic categorization. The predominant suggestions included optimizing utilization (34.88%) and reallocation (27.91%). Additional recommendations, albeit mentioned less frequently, encompassed storage, fixed asset management, disposal, manufacturer buyback, periodic verification, and donation.

## 4 Discussion

A major strength of this study lies in the establishment of an institution-level database that more accurately represents administrative hierarchies across China’s CDC system than individual-level data. Despite a 45% reduction in sample size due to stringent data screening, the high participation rate across multiple years ensures robust longitudinal insights and minimizes recall bias through respondent variation over time (10, 11).

Although the number of valid institutional datasets decreased from 239 in 2021 to 124 in 2023, this was primarily due to stricter data validation criteria and differences in completion rates, rather than sampling bias. The geographic coverage remained consistent across all 31 provinces, and the composition of CDC levels did not vary significantly, indicating minimal confounding in temporal trend analyses.

The analyses were conducted on anonymized, institution-level datasets collected independently each year; therefore, repeated participation of some CDCs cannot be fully excluded. Year-to-year comparisons should thus be interpreted as cross-sectional differences rather than longitudinal trajectories of identical institutions. Although this design limits causal inference, it accurately captures nationwide patterns in laboratory capacity during 2021–2023.

The study reveals that the penetration rate of nucleic acid testing equipment exceeds 95% across all levels of CDCs (Figure 1A), a likely consequence of the widespread deployment of nucleic acid testing hardware necessitated by extensive COVID-19 screening efforts (12). It is noteworthy that the CDC system in China has nearly achieved complete automation of nucleic acid extraction processes. Although comparable international data is scarce, factors such as reagent shortages, which disrupt supply chains, along with limited resources, high costs, and decreased operational flexibility, have necessitated the continued use or adaptation of manual extraction methods in some contexts (13, 14). The widespread implementation of Automated Nucleic Acid Extractors has significantly minimized human errors in sample processing, thereby enhancing both diagnostic accuracy and testing capacity (15). This automation further facilitates a higher yield of viral reads, even when employed in downstream applications such as metagenomic sequencing (16).

HTS, an indispensable tool for SARS-CoV-2 whole genome sequencing (WGS), had become widely adopted in provincial CDCs by 2021. The subsequent procurement of additional Bioinformatics Servers in 2022 was anticipated, as 6 of the top 10 technological buzzwords in 2021 pertained to bioinformatics analysis, reflecting the growing emphasis on computational capabilities in genomic research (5). Amidst China’s ongoing surveillance of SARS-CoV-2 genomic variations, municipal CDCs have augmented their HTS capacity over the past two years, which may have enhanced testing flexibility and throughput, potentially reducing manual workloads; however, these operational impacts were not directly quantified in this study. We foresee that municipal CDCs will further expand their acquisition of Bioinformatics Servers to enhance their capacity for “dry lab” activities. This shift is critical, as standardized bioinformatics quality control and data processing protocols are essential for ensuring the consistency and reliability of genomic analysis outcomes (17, 18).

In 2022, provincial CDCs enhanced their capabilities by acquiring additional pipetting workstations, significantly improving their ability to process large volumes of samples rapidly, particularly pooled samples. This acquisition not only amplifies testing capacity but also enhances diagnostic accuracy and consistency by minimizing human error, thus ensuring high standards of quality control in testing procedures (19, 20). As WGS transitions from a primarily research-focused tool to a standard diagnostic practice, the automation of labor-intensive library preparation using pipetting workstations offers a substantial reduction in manual handling time, while simultaneously maintaining high reliability and reproducibility (21). Alternatively, automation can be achieved through the integration of microfluidic platforms, further enhancing efficiency and precision in diagnostic workflows (22).

Higher-tier CDCs are typically equipped with more research-oriented equipment, with disparities more pronounced between municipal and county levels than between provincial and municipal levels. In 2022, provincial and municipal CDCs acquired ultracentrifuges, enhancing their capacity to isolate and purify viral particles or proteins, thereby facilitating the elucidation of viral replication and infection mechanisms (23). dPCR machines augment the analytical capabilities of Micro-Labs, enabling precise detection of low-concentration samples and low-frequency mutations. This technological advancement lays a critical foundation for identifying pathogen resistance mutations, monitoring viral load in HIV treatment, and analyzing genomic copy number variations (24).

Higher-tier CDCs are characterized by a significantly greater number of PCR Labs, BSCs, and qPCR machines, establishing the upper threshold of nucleic acid testing capabilities. The adequate provision of PCR Labs and BSCs as of 2021 suggests that the foundational infrastructure of these CDCs was sufficient to support subsequent enhancements, such as the acquisition of additional qPCR machines in 2022. We have delineated comprehensive reference standards and ranges for these three key facilities, derived from extensive empirical data, which reflect the average capabilities across various CDC levels and provide a robust benchmark for resource allocation and strategic planning. The recommended configurations derived from this study reflect empirically typical rather than prescriptive standards. They are proposed as reference benchmarks to support evidence-based planning, with adaptation to regional resources, financial constraints, and workforce capacity.

qPCR machines and HTS garner significant attention and are frequently discussed within the industry, owing to their indispensable roles, substantial costs, diverse options, and their direct impact on the outcomes of pandemic control efforts. Although numerous studies have conducted comparative analyses of performance parameters and consistency across different brands and models of qPCR machines and HTS (25, 26), data on their penetration rates and user satisfaction metrics remain scarce. It is noteworthy that the stringent data selection criteria employed in this study ensured that the 11 qPCR and 9 HTS models evaluated possessed a significant reputation and user base. As a result, even those models ranked lower are recognized for their commendable presence in the market. Although models with fewer than 10 ownership reports were excluded to maintain analytical reliability, this approach may underrepresent newer equipment types that have not yet achieved broad distribution.

The QA brand dominates the qPCR landscape, with three of its models securing the top positions in both penetration rate and user satisfaction, thereby affirming its status as the preeminent qPCR platform. QB and QC occupy the fourth and fifth positions respectively, each represented by a flagship model with a considerable user base. Notably, the sixth to ninth positions are held by domestic qPCR machine models, indicative of a robust multi-brand growth trajectory, as exemplified by qE, which has earned a strong reputation for quality (Figure 1B).

SA1 exhibits a commanding competitive advantage, consistently ranking first across multiple performance metrics. Its counterparts, SA2 and SA3, further diversify its application range, enriching its utilization across various contexts. The domestic sB1 has gained a substantial user base and positive reputation, signaling significant progress in China’s sequencing industry. Despite entering the market later, SC has rapidly gained traction, with its novel technical features earning it increasing adoption and recognition among CDCs (Figure 1C).

qPCR machines are renowned for their robust compatibility with a wide spectrum of reagents and consumables, a testament to their advanced technological maturity. This versatility has led to a proliferation of brands and models available in the market. Despite this diversity, several platform-specific considerations warrant careful attention. Foremost among these is the need for diagnostic kit development to align with leading qPCR brands and models, thereby ensuring broader market appeal and enhancing customer acquisition potential. Secondly, the utilization of multiple and distinct fluorescence channels by different qPCR platforms imposes additional requirements for signal calibration and consistent interpretation of results (27). This necessitates rigorous standardization to ensure analytical reliability across diverse experimental settings. Thirdly, contemporary qPCR machines have evolved to support modular conversion and functional expansion, enabling seamless integration with other laboratory equipment (28). As a result, qPCR machines are poised to serve as multifaceted tools within Micro-Labs, enhancing their versatility and broadening their applications in future diagnostic and research endeavors.

In contrast to the compatibility and convergence characteristic of qPCR machines, HTS library preparation and sequencing kits are notably more closed and exclusive. This exclusivity arises from the fundamental differences in sequencing principles among various HTS platforms, which dictate their applicability to distinct sequencing scenarios. For instance, a single Micro-Lab may require both next-generation sequencing (NGS) and third-generation sequencing (TGS) technologies to address tasks that prioritize either accuracy or timeliness. Additionally, these platforms may be employed collaboratively within the same research study to leverage their respective strengths (29). The rapid increase in domestic HTS adoption among municipal CDCs may reflect multiple factors, including evolving national procurement strategies and potential concerns about reagent supply stability or data security—issues that warrant further study rather than definitive attribution. This diversification likely enhanced local sequencing capacity and resilience during the transition from emergency response to routine genomic surveillance.

The shift from the vigorous emergency response to COVID-19 towards a more stabilized and routine surveillance framework inevitably results in an overabundance of Micro-Labs equipment. To address this, sharing laboratory equipment and even physical space can significantly reduce operational costs and enhance the utilization of laboratory resources. Furthermore, fostering collaboration and establishing sharing agreements between departments and institutions can mitigate the issue of equipment redundancy and idleness. The resale of idle equipment has been demonstrated as an effective strategy for resource reallocation, allowing budget-constrained laboratories to realize savings of 60%–90% by purchasing second-hand equipment (30). However, as government-sponsored public welfare institutions, CDCs must implement robust systems for equipment resale, compare to those for procurement, to ensure the rational allocation of resources while safeguarding against the potential loss of state assets. This requires a well-defined framework for implementation and oversight to balance resource optimization with the protection of public assets.

The trove of data amassed from three consecutive years of nationwide surveys in this field is exceptionally valuable, serving as a critical asset for future research and policy-making. This study has meticulously established a foundational framework and baseline for the hardware capabilities of CDC Micro-Labs, allowing CDC leadership and laboratory managers to conduct rigorous self-assessments against national benchmarks. Furthermore, these findings offer a strategic blueprint for the development and refinement of new Micro-Labs.

The systematic evaluation of user satisfaction across specific brands and models offers a rare and invaluable metric, providing essential insights that can guide CDCs in making informed purchasing decisions and driving manufacturers toward product optimization and refinement. The data underscores a robust and promising ascent of domestic brands, reflecting ongoing improvements in quality and performance. Ongoing progress in domestic equipment manufacturing suggests a trajectory toward higher competitiveness and greater alignment with international performance standards, though continued technological validation and benchmarking remain necessary. These developments collectively highlight China’s growing capacity to contribute high-quality, cost-effective solutions to global public health laboratory infrastructure. All interpretations should be viewed within the descriptive nature of this survey; causal relationships and operational impacts require validation through targeted analytical or experimental studies.

This study has several limitations. Although equipment data were verified through institutional records, self-reported information may still include inaccuracies. Satisfaction ratings are subjective and may reflect user familiarity or perception rather than objective performance. Excluding low-penetrance models improved statistical stability but may have underrepresented emerging technologies. The study did not assess equipment maintenance or operational performance, which could further influence laboratory capacity. As the survey focused solely on CDC laboratories, the findings may not fully represent hospital or private sectors. Variation in yearly participation may introduce nonresponse bias, although consistent geographic coverage suggests limited impact. These factors should be considered when interpreting the results and highlight the need for future on-site validation.

## Data Availability

The raw data supporting the conclusions of this article will be made available by the authors, without undue reservation.

## References

[1] Hunsperger E Juma B Onyango C Ochieng JB Omballa V Fields BS . Building laboratory capacity to detect and characterize pathogens of public and global health security concern in Kenya. BMC Public Health. (2019) 19:477. doi: 10.1186/s12889-019-6770-9, 32326916 PMC6696698

[2] Olver P Bohn MK Adeli K. Central role of laboratory medicine in public health and patient care. Clin Chem Lab Med. (2023) 61:666–73. doi: 10.1515/cclm-2022-1075, 36436024

[3] Lippi G Plebani M. The critical role of laboratory medicine during coronavirus disease 2019 (Covid-19) and other viral outbreaks. Clin Chem Lab Med. (2020) 58:1063–9. doi: 10.1515/cclm-2020-0240, 32191623

[4] Tong MX Hansen A Hanson-Easey S Xiang J Cameron S Liu Q . Public health professionals' perceptions of the capacity of China's Cdcs to address emerging and re-emerging infectious diseases. J Public Health (Oxf). (2021) 43:209–16. doi: 10.1093/pubmed/fdz070, 31251367

[5] Wang J Niu P Zhang R Li J Nie M Ma X. Current status and capacity of pathogen Laboratories in Centers for disease control and prevention in China during the Covid-19 pandemic: a Nationwide cross-sectional survey. Front Public Health. (2022) 10:927318. doi: 10.3389/fpubh.2022.927318, 36033752 PMC9404298

[6] Chauhan A Jindal T. Equipments and instruments for microbiological laboratories In: Chauhan A Jindal T, editors. Microbiological methods for environment, food and pharmaceutical analysis. Cham: Springer International Publishing (2020). 73–85.

[7] Gauthier NPG Chorlton SD Krajden M Manges AR. Agnostic sequencing for detection of viral pathogens. Clin Microbiol Rev. (2023) 36:e0011922. doi: 10.1128/cmr.00119-22, 36847515 PMC10035330

[8] Li C Sun M Wang Y Luo L Yu M Zhang Y . The Centers for Disease Control and Prevention system in China: trends from 2002-2012. Am J Public Health. (2016) 106:2093–102. doi: 10.2105/ajph.2016.303508, 27831781 PMC5105031

[9] Lasley JN Appiah EO Kojima K Blacksell SD. Global veterinary diagnostic laboratory equipment management and sustainability and implications for pandemic preparedness priorities. Emerg Infect Dis. (2023) 29:1–12. doi: 10.3201/eid2904.220778, 36958021 PMC10045690

[10] Niu P Sun Z Zhang R Zhao Y Tian F Cheng P . The state of biosafety across China's Cdc microbiology laboratories: insights from a Nationwide survey (2021–2023). Front Public Health. (2024) 12. doi: 10.3389/fpubh.2024.1436503, 39157525 PMC11327048

[11] Rettig T Blom AG. 3memory effects as a source of Bias in repeated survey measurement In: Cernat A Sakshaug JW, editors. Measurement error in longitudinal data: Oxford, United Kingdom: Oxford University Press (2021). 01.

[12] Fang Y. Large-scale National Screening for coronavirus disease 2019 in China. J Med Virol. (2020) 92:2266–8. doi: 10.1002/jmv.26173, 32533773 PMC7323343

[13] Miranda JP Osorio J Videla M Angel G Camponovo R Henríquez-Henríquez M. Analytical and clinical validation for Rt-Qpcr detection of Sars-Cov-2 without Rna extraction. Front Med. (2020) 7:567572. doi: 10.3389/fmed.2020.567572, 33178714 PMC7593567

[14] Barza R Patel P Sabatini L Singh K. Use of a simplified sample processing step without Rna extraction for direct Sars-Cov-2 Rt-Pcr detection. J Clin Virol. (2020) 132:104587. doi: 10.1016/j.jcv.2020.104587, 32898817 PMC7418644

[15] Dhibika M Madhusudhan NS Malini A Natarajan M. Comparison of manual and automated nucleic acid (Rna) extraction methods for the detection of Sars-Cov-2 by Qrt-Pcr. Cureus. (2023) 15:e36773. doi: 10.7759/cureus.36773, 37123735 PMC10133768

[16] Sabatier M Bal A Destras G Regue H Quéromès G Cheynet V . Comparison of nucleic acid extraction methods for a viral metagenomics analysis of respiratory viruses. Microorganisms. (2020) 8. doi: 10.3390/microorganisms8101539, 33036303 PMC7601816

[17] Wang S Niu P Su Q He X Tang J Wang J . Genomic surveillance for Sars-Cov-2—China, September 26, 2022 to January 29, 2023. China CDC Weekly. (2023) 5:143–51. doi: 10.46234/ccdcw2023.026, 37009519 PMC10061763

[18] Zufan SE Lau KA Donald A Hoang T Foster CSP Sikazwe C . Bioinformatic investigation of discordant sequence data for Sars-Cov-2: insights for robust genomic analysis during pandemic surveillance. Microb Genom. (2023) 9. doi: 10.1099/mgen.0.001146, 38019123 PMC10711311

[19] Mahase E. Covid-19: universities roll out pooled testing of students in bid to keep campuses open. BMJ (Clinical research ed). (2020) 370:m3789. doi: 10.1136/bmj.m3789, 32994203

[20] Matsumura Y Noguchi T Shinohara K Yamamoto M Nagao M. Development and evaluation of three automated media pooling and molecular diagnostic systems for the detection of Sars-Cov-2. Microbiol Spectr. (2024) 12:e0368423. doi: 10.1128/spectrum.03684-23, 38289934 PMC10913432

[21] Meijers E Verhees FB Heemskerk D Wessels E Claas ECJ Boers SA. Automating the Illumina DNA library preparation kit for whole genome sequencing applications on the Flowbot one liquid handler robot. Sci Rep. (2024) 14:8159. doi: 10.1038/s41598-024-58963-2, 38589623 PMC11001922

[22] Hoffmann A Timm A Johnson C Rupp S Grumaz C. Automation of customizable library preparation for next-generation sequencing into an open microfluidic platform. Sci Rep. (2024) 14:17150. doi: 10.1038/s41598-024-67950-6, 39060329 PMC11282295

[23] Yao H Song Y Chen Y Wu N Xu J Sun C . Molecular architecture of the Sars-Cov-2 virus. Cell. (2020) 183:730–8.e13. doi: 10.1016/j.cell.2020.09.018, 32979942 PMC7474903

[24] Kojabad AA Farzanehpour M Galeh HEG Dorostkar R Jafarpour A Bolandian M . Droplet digital Pcr of viral DNA/Rna, current Progress, challenges, and future perspectives. J Med Virol. (2021) 93:4182–97. doi: 10.1002/jmv.26846, 33538349 PMC8013307

[25] Dellière S Gits-Muselli M White PL Mengoli C Bretagne S Alanio A. Quantification of *pneumocystis jirovecii*: cross-platform comparison of one Qpcr assay with leading platforms and six master mixes. J Fungi (Basel). (2019) 6. doi: 10.3390/jof6010009, 31888050 PMC7151141

[26] Pecman A Adams I Gutiérrez-Aguirre I Fox A Boonham N Ravnikar M . Systematic comparison of Nanopore and Illumina sequencing for the detection of plant viruses and Viroids using Total Rna sequencing approach. Front Microbiol. (2022) 13:883921. doi: 10.3389/fmicb.2022.883921, 35633678 PMC9131090

[27] Zhang H Yan Z Wang X Gaňová M Chang H Laššáková S . Determination of advantages and limitations of Qpcr duplexing in a single Fluorescent Channel. ACS omega. (2021) 6:22292–300. doi: 10.1021/acsomega.1c02971, 34497918 PMC8412922

[28] Cereda M Cocci A Cucchi D Raia L Pirola D Bruno L . Q3: a compact device for quick, high precision Qpcr. Sensors (Basel). (2018) 18. doi: 10.3390/s18082583, 30087266 PMC6111627

[29] Xia Y Li X Wu Z Nie C Cheng Z Sun Y . Strategies and tools in Illumina and Nanopore-integrated metagenomic analysis of microbiome data. iMeta. (2023) 2:e72. doi: 10.1002/imt2.72, 38868337 PMC10989838

[30] Hickman R Nguyen J Lee TD Tyson JR Azana R Tsang F . Rapid, high-throughput, cost-effective whole-genome sequencing of Sars-Cov-2 using a condensed library preparation of the Illumina DNA prep kit. J Clin Microbiol. (2024) 62:e0010322. doi: 10.1128/jcm.00103-22 38315007 PMC11210267

