# Supplementary material for: Three-year nationwide survey of microbiology laboratory equipment conditions in China’s CDC: setting new benchmarks
Source: Front Public Health. 2026 Jan 13;13:1679072. doi: 10.3389/fpubh.2025.1679072 (PMC12835382; doi:10.3389/fpubh.2025.1679072)
Supplement: Supplementary file 1 [file Data_Sheet_1.docx]

**National Survey on the Equipment Conditions of Microbiology Laboratories in China CDCs**

**Section 1. Basic Information**

1. Name
2. Gender
3. Age
4. Mobile phone number
5. Professional title
6. Highest educational qualification / academic degree
7. Affiliated institution
8. Position / job title
9. Province

**Section 2. Questionnaire Content**

1. Multiple choice: Which biosafety level laboratories are currently established and operational in your institution? (Select all that apply)

A. BSL-1

B. BSL-2

C. BSL-3

D. BSL-4

E. None

2. Single choice: How many standard PCR laboratories for pathogen detection are available in your institution? (Note: Each laboratory should include at least three functional zones-reagent preparation, sample processing, and nucleic acid amplification.)

A. 1 laboratory

B. 2 laboratories

C. 3 laboratories

D. 4 laboratories

E. 5 laboratories

F. More than 5 laboratories

G. None

3. Multiple choice: Which of the following laboratory instruments are currently functional in your institution? (Select all that apply)

A. Autoclave (for decontaminating biohazardous waste)

B. Autoclave (for sterilizing clean laboratory consumables)

C. Biological safety cabinet (BSC)

D. Standard centrifuge

E. Refrigerated centrifuge

F. Pulsed-field gel electrophoresis system (PFGE)

G. Automated nucleic acid extraction system

H. ELISA reader / analyzer

I. –20℃ freezer

J. –70℃ freezer

K. Cell culture incubator

L. Cell counter

M. Conventional PCR thermocycler

N. Real-time quantitative PCR (qPCR) machine

O. Digital PCR (dPCR) machine

P. Sanger sequencing platform (first-generation sequencing)

Q. High-throughput sequencing platform (second- or third-generation sequencing)

R. Bioinformatics server

S. Pipetting workstation (for PCR system setup or sequencing library preparation)

T. Ultracentrifuge

4. Single choice: How many biological safety cabinets (BSCs) in your institution are available for pathogen detection?

A. 1 unit

B. 2 units

C. 3 units

D. 4 units

E. 5 units

F. 6 units

G. 7 units

H. 8 units

I. 9 units

J. ≥10 units

K. ≥20 units

5. Single choice: How many real-time quantitative PCR (qPCR) instruments are available in your institution for pathogen nucleic acid detection?

A. 1 unit

B. 2 units

C. 3 units

D. 4 units

E. 5 units

F. 6 units

G. 7 units

H. 8 units

I. 9 units

J. ≥10 units

K. ≥20 units

6. Multiple choice: Which of the following real-time quantitative PCR (qPCR) instruments are currently functional in your institution? (Select all that apply)

A. None

B. ABI 7300

C. ABI 7500

D. ABI 7900

E. ABI Q5

F. ABI Q7

G. Roche LightCycler 480

H. Bio-Rad CFX96

I. Bio-Rad iQ5

J. Daan Gene (China)

K. Hangzhou Bioer Technology (China)

L. Shanghai Hongshi (China)

M. Xi’an Tianlong (China)

N. Other (please specify)

7. Multiple choice: Among the qPCR instruments listed in Question 6, which ones do you consider to have a high level of user satisfaction? (Select all that apply)

A. None

B. ABI 7300

C. ABI 7500

D. ABI 7900

E. ABI Q5

F. ABI Q7

G. Roche LightCycler 480

H. Bio-Rad CFX96

I. Bio-Rad iQ5

J. Daan Gene (China)

K. Hangzhou Bioer Technology (China)

L. Shanghai Hongshi (China)

M. Xi’an Tianlong (China)

N. Other (please specify)

8. Multiple choice: Which of the following high-throughput sequencing platforms are currently functional in your institution? (Select all that apply)

A. None

B. Illumina iSeq

C. Illumina MiSeq

D. Illumina NextSeq

E. Roche 454

F. Thermo Fisher Ion Torrent

G. Thermo Fisher Ion S5

H. PacBio RS

I. Oxford Nanopore MinION / Mk1C

J. Oxford Nanopore GridION

K. MGI 200

L. MGI 2000

M. Illumina NovaSeq / BGI T7

N. Other (please specify)

9. Multiple choice: Among the high-throughput sequencing platforms listed in Question 8, which ones do you consider to have a high level of user satisfaction? (Select all that apply)

A. None

B. Illumina iSeq

C. Illumina MiSeq

D. Illumina NextSeq

E. Roche 454

F. Thermo Fisher Ion Torrent

G. Thermo Fisher Ion S5

H. PacBio RS

I. Oxford Nanopore MinION / Mk1C

J. Oxford Nanopore GridION

K. MGI 200

L. MGI 2000

M. Illumina NovaSeq / BGI T7

N. Other (please specify)

10. Open-ended question: Besides the equipment listed above, what other instruments do you consider important for microbiology laboratories in CDCs?
