# Supplementary figures and images for: Three-year nationwide survey of microbiology laboratory equipment conditions in China’s CDC: setting new benchmarks
Source: Front Public Health. 2026 Jan 13;13:1679072. doi: 10.3389/fpubh.2025.1679072 (PMC12835382; doi:10.3389/fpubh.2025.1679072)

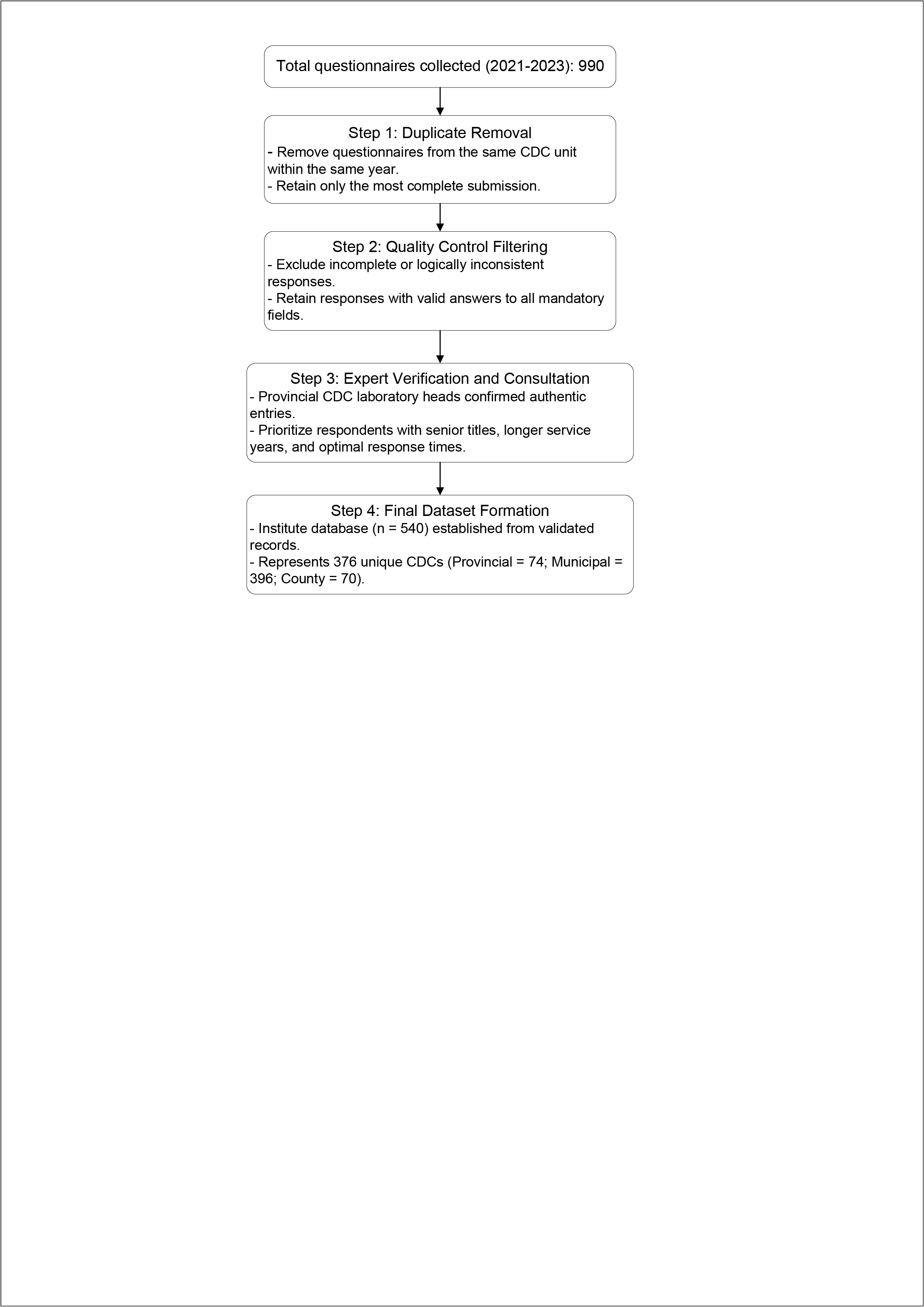

Supplement: Supplementary file 2 [file Image_1.jpg]
